# Shifting Enteric Fever Epidemiology in Children in Nepal: Is the Burden of *Salmonella* Paratyphi on the Rise?

**DOI:** 10.31729/jnma.v63i290.9200

**Published:** 2025-09-01

**Authors:** Shrijana Shrestha, Sanjeev Man Bijukchhe, Saugat Bhandari, Meeru Gurung

**Affiliations:** 1 Department of Paediatrics, Patan Academy of Health Sciences, Kathmandu, Nepal

**Keywords:** *children*, *enteric fever*, *Nepal*, *paratyphoid*, *typhoid conjugate vaccine*

## Abstract

Nepal has a high burden of enteric fevers. An observational study conducted in a tertiary-level hospital in Nepal over 11 years (2014-2024), reviewing hospital records of all blood culture-positive cases of *Salmonella* Typhi and Paratyphi in children, showed a gradual decline in blood culture-positive enteric fever cases with year-to-year variability. Typhoid conjugate vaccine (TCV) was introduced into the national immunisation schedule of Nepal in 2022. We observed that the proportion of *Salmonella* Paratyphi cases has increased in the post-typhoid conjugate vaccine years compared with the pre-typhoid conjugate vaccine period. A slight rise in hospitalisation rates for blood culture-positive paratyphoid infections has also been observed in the post-typhoid conjugate vaccine period. These findings underscore the importance of continued surveillance and call for further evaluation of the potential shift in enteric fever epidemiology. The development of effective vaccines targeting both typhoid and paratyphoid remains crucial for comprehensive disease control.

## INTRODUCTION

Enteric fever remains a major global health concern. In 2021, there were estimated 700,000 cases and 90,000 deaths due to enteric fevers worldwide.^1,2^ The advances in vaccination, clean water and sanitation have contributed to declining case numbers. Over the years, global incidence and mortality of enteric fever have decreased, yet children under five still accounted for 40% of deaths, with the highest case fatality in countries with low socio-demographic index.^3^ Among enteric fevers, paratyphoid accounts for around 20% of cases globally.^3^ Paratyphoid fever has been found responsible for a growing proportion of enteric fevers especially in South and South-East Asia.^4^

## THE CONTINUING BURDEN OF ENTERIC FEVER

Although vaccination coverage and improvements in water and sanitation have led to progress, enteric fever continues to pose a substantial global health burden, particularly in children under five years of age. Between 2017 and 2021, the age-standardised incidence rate of enteric fever declined from 152 (118-195) to 128 (100-163) cases per 100,000 person-years globally, while the mortality rate fell from 1.87 (0.95-3.18) to 1.50 (0.78-2.54) deaths per 100,000 person-years.^3^ Case fatality remained highest in countries with low socio-demographic index and, although there was an overall global decline, no reduction was observed among children aged 1-4 years.^3^

Nepal has a high burden of enteric fever. A passive surveillance conducted in Nepal between 2016 and 2018 showed a crude incidence of blood culture-positive enteric fever of 74 (62-87)/100,000 (95% CI) and adjusted incidence (adjusted for blood-culture sensitivity, probability of receiving a blood-culture and health-care seeking) of 1062(683-1839)/ 100,000 person years.^1^ Data from a sentinel surveillance site (Patan hospital, in Lalitpur district) for invasive bacterial diseases (IBD) in children showed that *Salmonella* Typhi accounted for 28% of all pathogens isolated from blood-culture and 1.6% of all blood-cultures performed in admitted children (2005 and 2013).^5^

As part of a long running invasive bacterial disease surveillance in children, the Paediatric Research Unit at Patan Academy of Health Sciences, Nepal, has maintained a record of all positive blood culture isolates since 2014, including *Salmonella* Typhi and *Salmonella* Paratyphi, from children 1 month to 15 years of age who underwent blood culture testing at Patan hospital. During the study period (2014-2024) a total of 717 cases of enteric fever were identified, of which 579 (80.75%) were positive for *Salmonella* Typhi and 138 (19.25%) for *Salmonella* Paratyphi.

## TREND OF ENTERIC FEVER CASES AND TCV INTRODUCTION IN NEPAL

Between January 2014 and December 2024, over the 11-year period a gradual decline in the blood culture-positive enteric fever cases was observed, with some year-to-year variability.

Nepal introduced Typhoid conjugate vaccine (TCV) into the National Immunization Programme in May, 2022 and became the fourth Gavi-supported country to introduce TCV in the routine immunization programme. Before the introduction of TCV into the NIP, a nationwide campaign targeting children 15 months to 15 years was conducted (April/May, 2022) with a very high (>90%) coverage.^6^

Blood culture-positive enteric fever cases from a median number of 86.5 /year (IQR 63-122) in the pre-COVID-19 and pre-TCV years (2014 to 2019), decreased to a median number of <20 cases/year during the years affected by COVID-19 pandemic (2020 – 2021). Post-COVID-19 pandemic and post-TCV introduction years (2022-2024), the number of cases increased slightly but remained under 50/year (median 48; IQR 47-50).

## ALTERNATIVE EXPLANATIONS FOR THE CHANGING TREND

Before the introduction of TCV in the National Immunization Programme, between 2017 and 2019, 20,000 children from the catchment area of the hospital in Lalitpur district received a dose of TCV, as part of a phase III clinical trial conducted by Patan Hospital in collaboration with University of Oxford, and this could have, to some extent, contributed to the decline in enteric fever cases presenting to Patan Hospital.^7^ In addition to TCV, several other factors could be contributory to this decline. Improvements in water, sanitation, and hygiene (WASH) interventions over the years could have played a major role. Changes in the health-seeking behaviour, improved healthcare access due to an increase in both public and mainly private health facilities within the district, and availability of over-the-counter antibiotics, have also contributed to this trend.

## RELATIVE BURDEN OF PARATYPHOID AMONG CULTURE-POSITIVE ENTERIC FEVER CASES

Among enteric fevers, paratyphoid accounts for around 20% of cases globally. It has been reported that the relative burden of typhoid and paratyphoid varies depending upon the geographical location; paratyphoid being most prevalent in South and SouthEast Asia.^4^ In some Asian countries. *S.* Paratyphi has been found responsible for a growing proportion of enteric fever cases.^8^ S. Paratyphi A, in particular, has been identified as the main causal agent in countries such as India and China which represent the world’s largest populations.^9^ Paratyphoid fever is reportedly on the rise and concerns have been raised that the increase may be associated with typhoid vaccination.^4^ With these concerns, the need for a bivalent vaccine, protecting against both typhoid and paratyphoid was recognized several years ago.^9^ Previous studies conducted at Patan hospital, including both children and adults showed *Salmonella* Paratyphi contributing to 29.3% (1993-2003)^10^ and 31% (2005-2009)^11^ of blood culture-positive enteric fevers. Over the 11 years period (2014-2024), at Patan Hospital we observed that the contribution of paratyphoid cases to the total blood culture-positive enteric fevers varied over the years, with the median of 12.5% (IQR 4.9-28).

## THE RISE OF PARATYPHOID CASES IN THE POST-TCV YEARS IN NEPAL

The relative contribution of *S.* Paratyphi among culture positive enteric fevers was observed to be 10.8% before the introduction of TCV into the national immunisation schedule of Nepal. In the year of TCV introduction and the catch-up campaign (2022), culture-positive paratyphoid cases accounted for 61% of total culture-positive enteric fevers. In the subsequent year (2023), the relative contribution of paratyphoid decreased to 20% but it rose again to 77% in 2024, the second post-TCV year, (highest in the 11-years observational period). The increase in the relative contribution of *S.* Paratyphi in the post-TCV period (52.4%; 76/145) compared with the pre-TCV period (10.8%;62/572) was statistically significant; (p<0.001). In this period, 17.5% culturepositive Enteric fever cases were hospitalized; 108/579 (18.6%) cases of S. Typhi, and 20/138 (14.5%) cases of *S.* Paratyphi. Comparing hospitalization rates in pre-TCV and post-TCV periods, a slight increase was observed in the post-TCV period (17.3% vs 20%; p=0.45), the increase contributed mainly by Paratyphi. Hospitalization rate for paratyphoid increased from 8% (5/62) in the pre-TCV to 19.7% (15/76) in the post-TCV period; the increase was not statistically significant (p=0.053).

## FUTURE DIRECTIONS FOR SURVEILLANCE AND RESEARCH

The rising relative burden of paratyphoid, particularly in South and South-East Asia, emphasizes the need for enhanced surveillance and targeted research. Future research should prioritise understanding drivers of paratyphoid transmission, evaluating the impact of TCV, and advancing development of paratyphoid vaccines or bivalent vaccines covering both typhoid and paratyphoid to ensure comprehensive protection against *S.* Typhi and *S.* Paratyphi. Our findings are limited to a single centre, and the post-TCV data covers only a short period (three years). To strengthen the evidence base, future research should be conducted across multiple centres and diverse geographical settings, with longer follow-up periods, post-TCV introduction, to capture broader epidemiological trends and better understand the evolving burden of both typhoid and paratyphoid fever.

## CONCLUSIONS

Enteric fever continues to be a significant cause of fever in Nepali children. The shifting epidemiology of enteric fever with a change in the relative burden of paratyphoid cases raises concern. Our findings are consistent with the reported observations on the increasing paratyphoid cases following vaccination against typhoid. These observations highlight the need for sustained surveillance and development of effective vaccines against paratyphoid or bivalent vaccines targeting both typhoid and paratyphoid fevers.
